# Supplementary material for: Computational Workflow to Study the Diversity of Secondary Metabolites in Fourteen Different Isatis Species
Source: Cells. 2022 Mar 6;11(5):907. doi: 10.3390/cells11050907 (PMC8909408; doi:10.3390/cells11050907)
Supplement: Supplementary file 1 [file cells-11-00907-s001.zip › Supplementary file S13. Tutorial of MetaboFR/Tutorial for MetaboFR.pdf]

The workflow of MetaboFR tool

Step 1:

Software installation:

MS-DIAL:
http://prime.psc.riken.jp/Metabolomics_Software/MS-DIAL/index2.html
R version up to 3.6.1 : https://cran.r-project.org/
R studio: https://rstudio.com/products/rstudio/

Step 2:

MSCleanR package installation: (*Anal. Chem*. 2020,92(14):9971-9981**)**

```
install.packages("devtools")
library(devtools)
library(usethis)
devtools::install_github("eMetaboHUB/MS-CleanR")
library(mscleanr)
runGUI()
```

Step 3:

Prepare adduct ions files for MetaboFR:

Create “adduct_pos.csv” or “adduct_neg.csv” files

Insert different adduct types in Column “A” and “B”

Calculate mass difference between different adduct types for one adduct correlation.

Insert accurate mass different in Column “C”

Case: adduct_pos.csv

	A	B	C
1	initial	final	diff
2	M+H	M+Na	21.98194
3	M+H	M+NH4	17.02655
4	M+H	M+K	37.95588
5	M+NH4	M+Na	4.955395
6	M+NH4	M+K	20.92934
7	M+Na	M+K	15.97394

Each row means one adduct correlation

Step 4:

Run MetaFR package:

Run CleanR to produce “MS_peaks-clusters_final” file

Put “adduct ions files” and “MS_peaks-clusters_final” in the same folder

Run Rstudio software: File-Open project- Open “ChemicalDupCK ” in MetaFR folder

-Click “RetypeDupChk.r”-Choose “MS_peaks-clusters_final”

-Produce “res_dupChkXXXXXXXX”

Notes: res_dupChkXXXXXXXX: XXXXXXXX means the date of the file produced

The operational guidance of MetaboFR tool

1

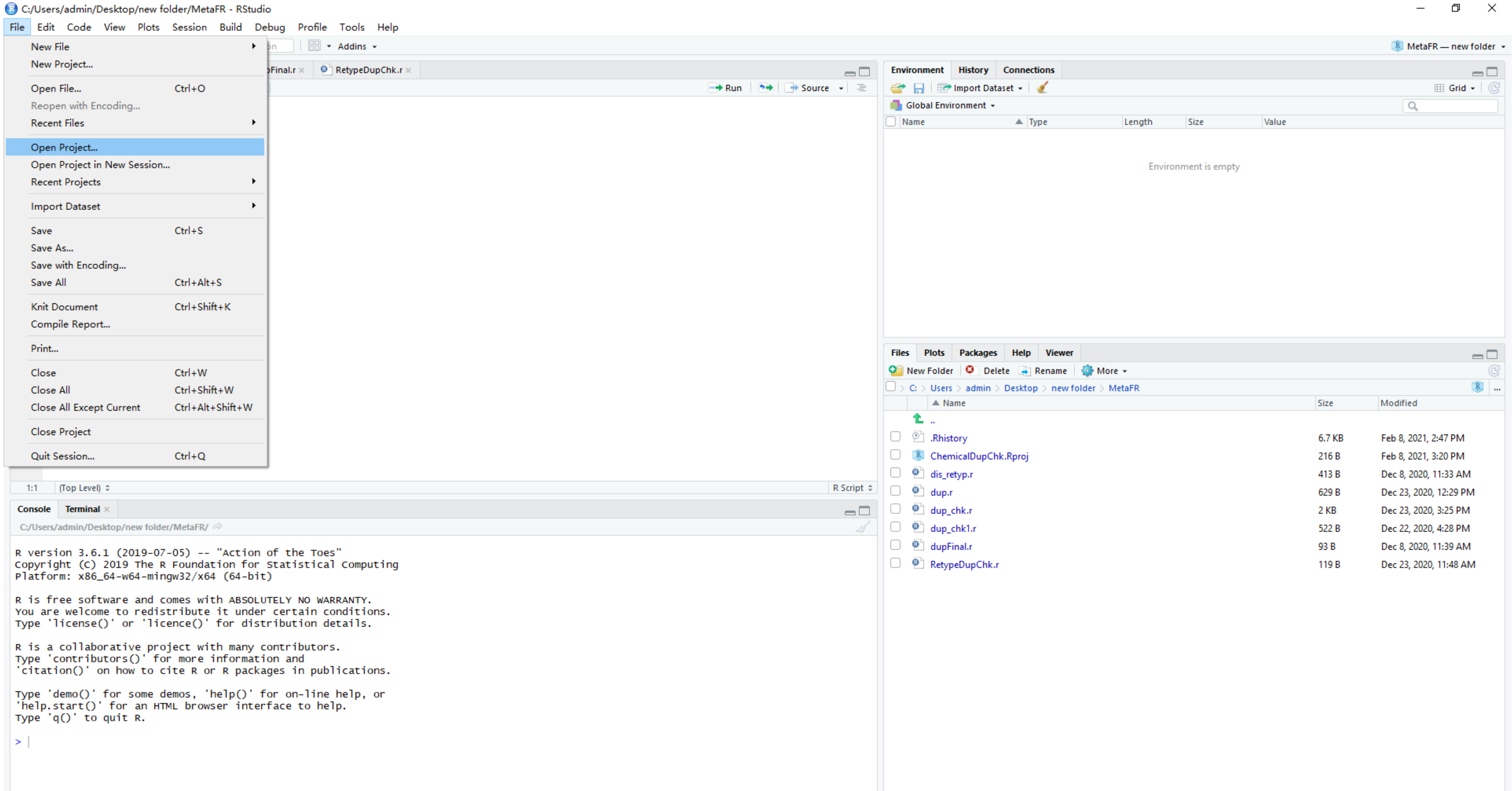

Open R-studio and select 'file' menu, then click 'Open Project' with 'ChemicalDupChk' in MetaboFR folder.

2

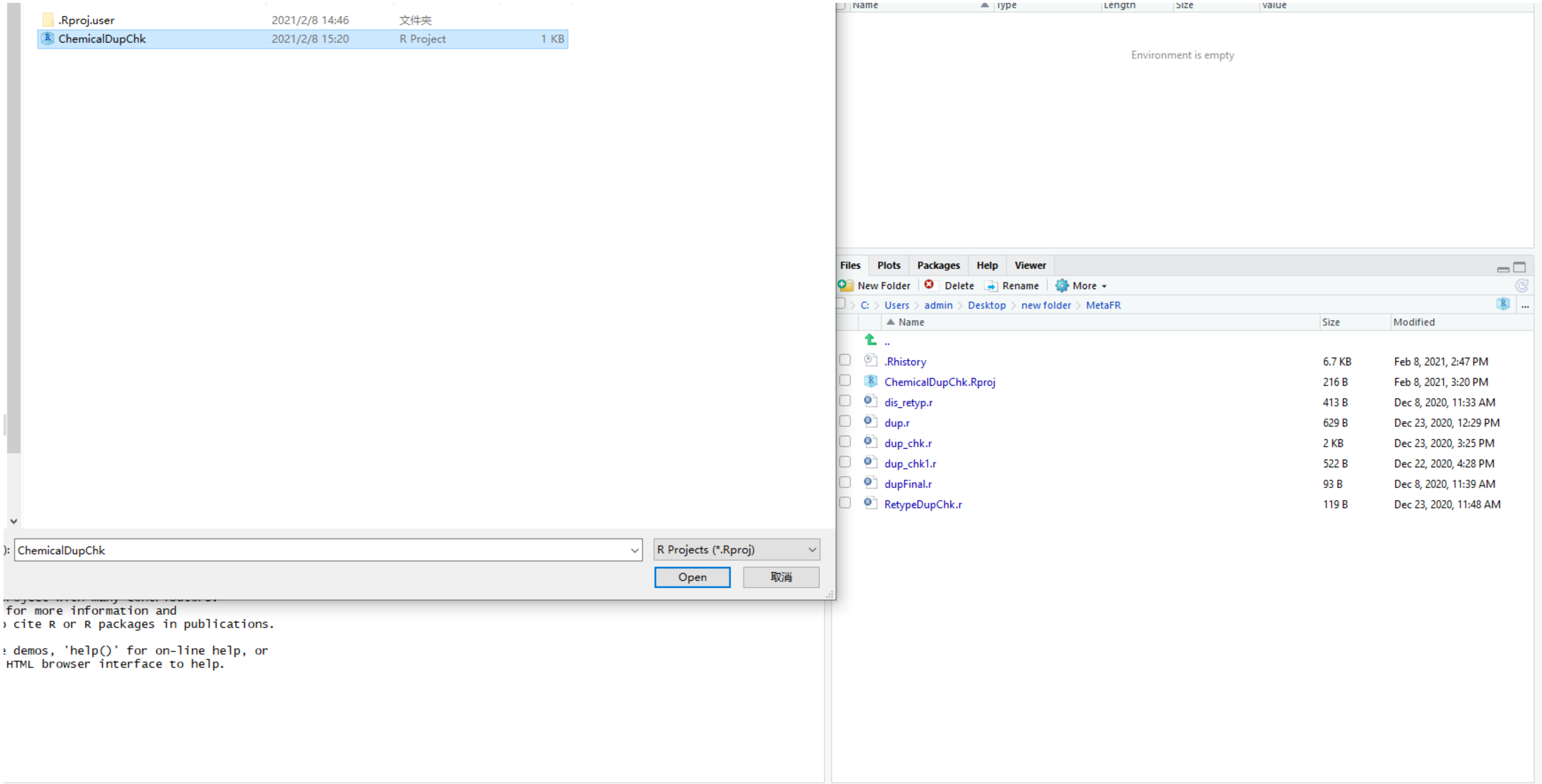

Open R-studio and select 'file' menu, then click 'Open Project' with 'ChemicalDupChk' in MetaboFR folder.

3

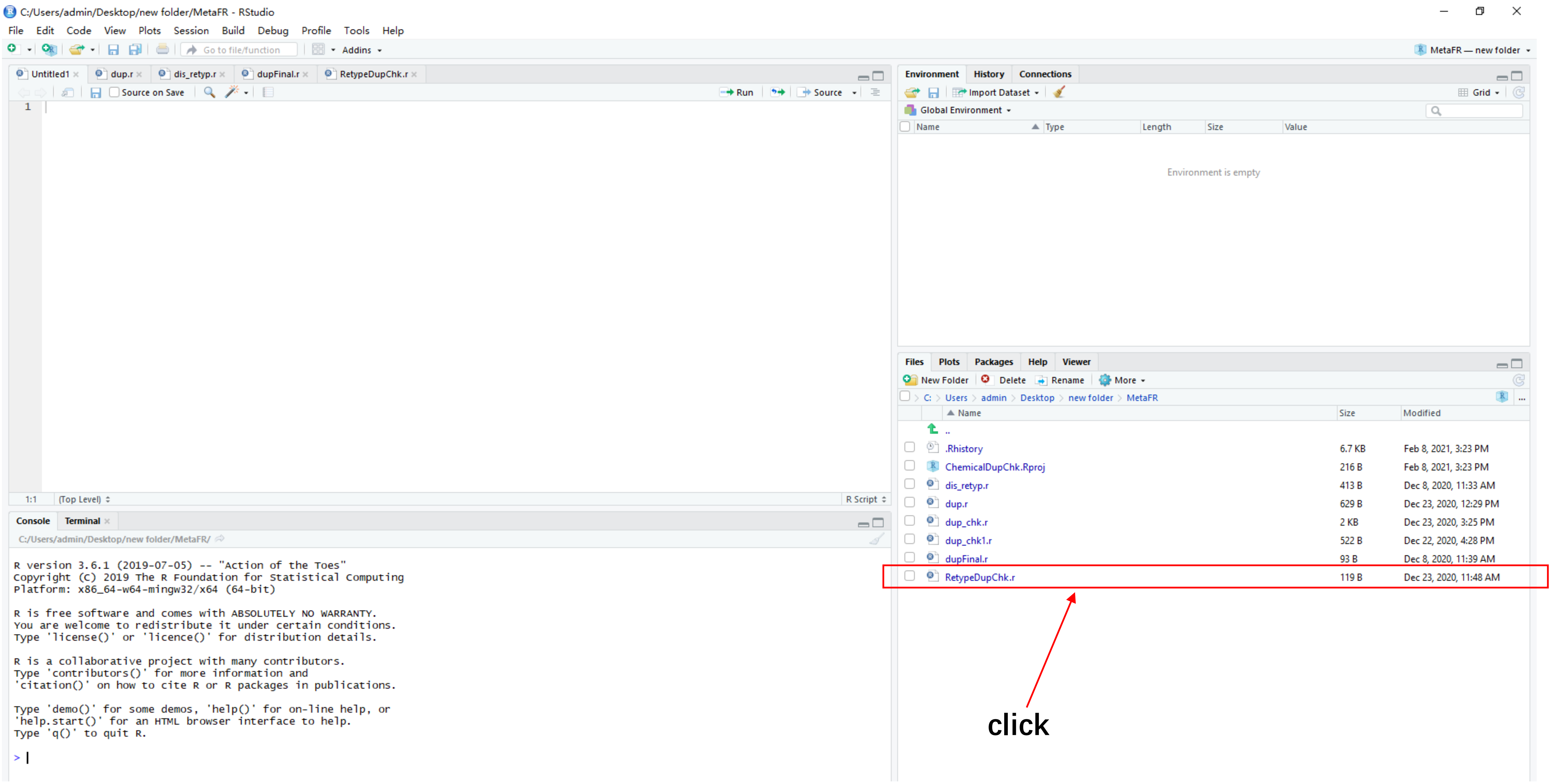

Then click 'RetypeDupChk.r' in 'ChemicalDupChk' project.

4

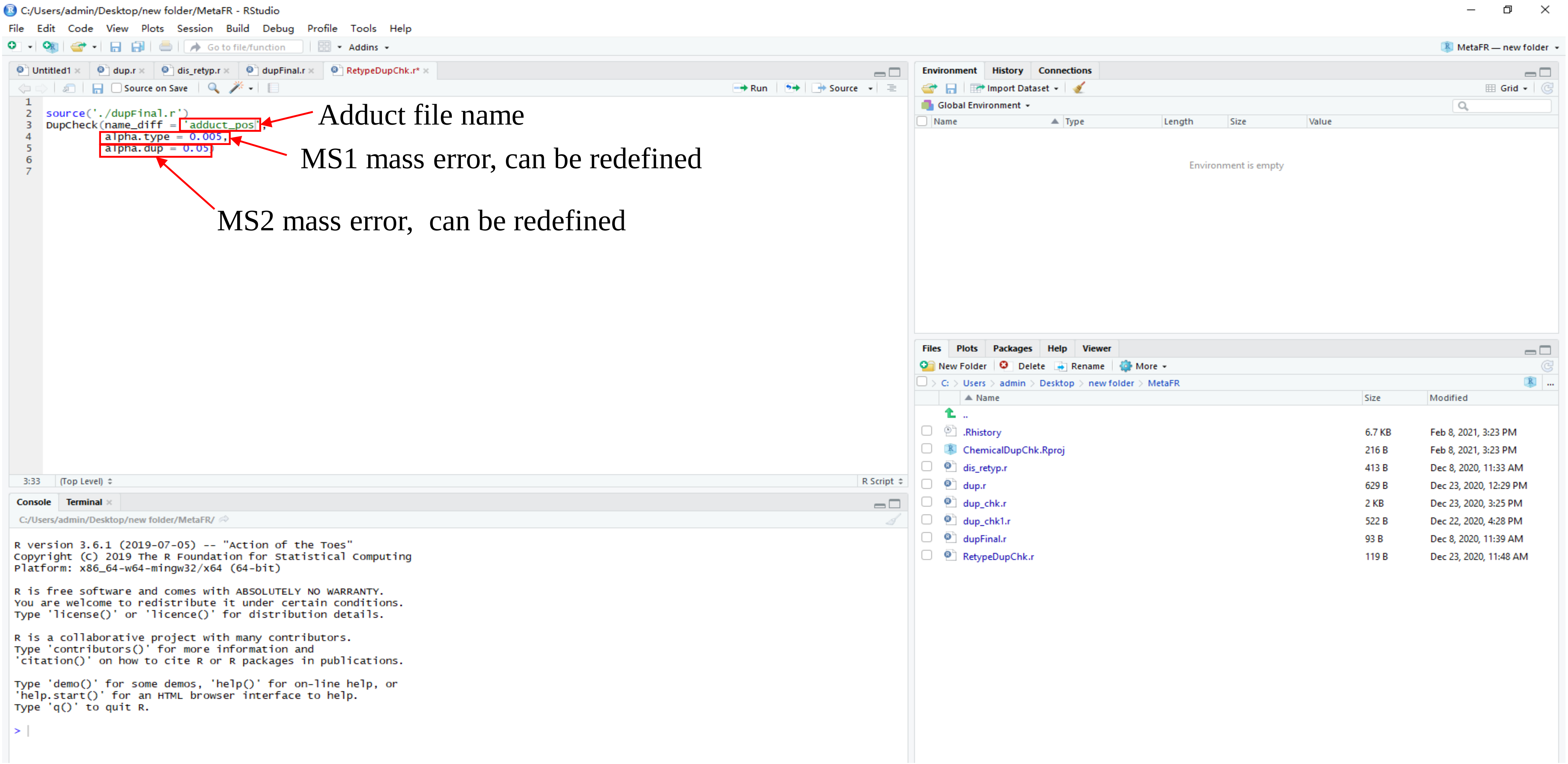

After that, you can run the program, in which 'adduct file name', 'MS1 mass error', and 'MS2 mass error' can be defined according to your request.

5

MetaFR	2021/2/8 15:23	文件夹	
adduct_neg	2021/1/4 12:16	Microsoft Excel ...	1 KB
adduct_pos	2020/12/16 11:05	Microsoft Excel ...	1 KB
res_dupChk20210103	2021/1/3 13:20	Microsoft Excel ...	204 KB

Finally, you will obtain the processed data, named ‘res_dupChk XXXXX’.

6

The peak table processed by MSCleanR (4072 features)

Cluster number — MS1 *m/z* — MS2 fragment

A	B	C	D	E	F	G	H	I	J	K	L	M	N	O	P	Q
1569	20	1479	22	0.00368	pos_454(	pos	4540	4.41	787.229	[M+H]+	adduct]	0.425	595.05	isa1-pos	787.22638:	103.0539
1569	20	1479	22	0	pos_304	pos	304	4.423	174.113	[M+H]+	ion corr	0.138	116.37	isa1-pos	174.1125:1	102.0372
1569	20	1479	22	0	pos_445	pos	445	4.467	193.086	[M+H]+	ion corr	0.075	87.87	isa6-pos	193.0863:2	103.0535
1569	20	1479	22	0	pos_322	pos	322	4.435	176.071	[M+H]+	ion corr	0.013	173.87	isa7-pos	176.07101:	104.0508
1569	20	1479	22	0	pos_162(	pos	1620	4.447	327.157	[M+H]+	ion corr	0.013	45.49	isa8-pos	327.15704:	103.0523
1569	20	1479	22	0	pos_461(	pos	4610	4.417	809.21	[M+H]+	NA	0.262	155.51	isa2-pos	809.20862:	104.0503
1569	20	1479	22	0	pos_3391	pos	3391	4.425	527.174	[M+H]+	NA	0.087	83.73	isa3-pos	527.17279:	102.0469
1569	20	1479	22	0	pos_1294	pos	1294	4.464	298.098	[M+H]+	NA	0.988	506.48	isa1-pos	298.09717:	104.0504
1569	20	1479	22	0	pos_825	pos	825	4.435	246.124	[M+H]+	NA	0.013	121.87	isa7-pos	246.12413:	104.0508
1569	20	1479	22	0	pos_2906	pos	2906	4.442	447.14	[M+H]+	NA	0.075	64.41	isa7-pos	447.13757:	103.0531
1569	20	1479	22	0	pos_2714	pos	2714	4.442	425.085	[M+H]+	NA	0.025	81.57	isa13-pc	425.08408:	107.9669
1569	20	1479	22	0	pos_2738	pos	2738	4.439	426.129	[M+H]+	similar	0.175	120.92	isa1-pos	426.12927:	104.0499
1569	20	1479	22	0	pos_1912	pos	1912	4.442	351.119	[M+H]+	similar	0.363	142.82	isa2-pos	351.11829:	102.0386
1569	20	1479	22	0.00378	pos_2584	pos	2584	4.482	411.109	[M+K]+	adduct]	0.087	39.48	isa6-pos	411.10898:	103.0544
1569	20	1479	22	0.00378	pos_4344	pos	4344	4.436	725.239	[M+K]+	adduct]	0.013	136.36	isa8-pos	725.23926:	103.0524
1569	20	1479	22	0.00368	pos_4632	pos	4632	4.357	825.181	[M+K]+	adduct]	0.013	235.95	isa4-pos	825.18146:	101.0232
1569	20	1479	22	0.00585	pos_2414	pos	2414	4.473	395.132	[M+Na]+	adduct]	0.188	107.92	isa2-pos	395.1304:8	104.0501
1569	20	1479	22	0.00585	pos_4313	pos	4313	4.459	709.268	[M+Na]+	adduct]	0.525	185.64	isa2-pos	709.26483:	104.0501
1569	20	1479	22	0.00963	pos_4295	pos	4295	4.447	704.31	[M+NH4]+	ion corr	0.013	109.84	isa8-pos	704.30981:	103.0523
1569	20	1479	22	0.00963	pos_2355	pos	2355	4.467	390.176	[M+NH4]+	ion corr	0.087	102.54	isa6-pos	390.17575:	103.0535

20 features in this cluster

Original peak table obtained by MSCleanR: Cluster-1569 contained 20 features in it.

7

The obtained peak table processed by MetaboFR (3426 features)

Adduct flagging

A	B	C	D	E	F	G	H	I	J	K	L	M	N	O	P	Q	EU
1569	20	1479	22	0.00368	pos_4632	pos	4632	4.357	825.181	[M+K]+	SRF (Unl	0.013	235.95	isa4-pos	825.1814	101.0232	M+K
1569	20	1479	22	0.00368	pos_4540	pos	4540	4.41	787.229	[M+H]+	FRGM	0.425	595.05	isa1-pos	787.2263	103.0539	M+H
1569	20	1479	22	0	pos_4610	pos	4610	4.417	809.21	[M+H]+	FRGM	0.262	155.51	isa2-pos	809.2086	104.0503	M+Na
1569	20	1479	22	0	pos_3391	pos	3391	4.425	527.174	[M+H]+	SRF (ID)	0.087	83.73	isa3-pos	527.1727	102.0469	
1569	20	1479	22	0	pos_825	pos	825	4.435	246.124	[M+H]+	SRF (ID)	0.013	121.87	isa7-pos	246.1241	104.0508	
1569	20	1479	22	0.00378	pos_4344	pos	4344	4.436	725.239	[M+K]+	TRF	0.013	136.36	isa8-pos	725.2392	103.0524	M+K
1569	20	1479	22	0	pos_1912	pos	1912	4.442	351.119	[M+H]+	SRF (ID)	0.363	142.82	isa2-pos	351.1182	102.0386	
1569	20	1479	22	0	pos_2714	pos	2714	4.442	425.085	[M+H]+	SRF (Unl	0.025	81.57	isa13-pc	425.0840	107.9669	
1569	20	1479	22	0.00963	pos_4295	pos	4295	4.447	704.31	[M+NH4]+	TRF (mos	0.013	109.84	isa8-pos	704.3098	103.0523	M+NH4
1569	20	1479	22	0.00585	pos_4313	pos	4313	4.459	709.268	[M+Na]+	TRF	0.525	185.64	isa2-pos	709.2648	104.0501	M+Na
1569	20	1479	22	0	pos_1294	pos	1294	4.464	298.098	[M+H]+	SRF (ID)	0.988	506.48	isa1-pos	298.0971	104.0504	
1569	20	1479	22	0.00963	pos_2355	pos	2355	4.467	390.176	[M+NH4]+	TRF (mos	0.087	102.54	isa6-pos	390.1757	103.0535	M+NH4
1569	20	1479	22	0.00585	pos_2414	pos	2414	4.473	395.132	[M+Na]+	TRF	0.188	107.92	isa2-pos	395.1304	104.0501	M+Na
1569	20	1479	22	0.00378	pos_2584	pos	2584	4.482	411.109	[M+K]+	TRF	0.087	39.48	isa6-pos	411.1089	103.0544	M+K

After processed by MetaboFR (14 features in this cluster)

Second-rated features (SRFs)
No adducts

Top-rated features (TRFs)
Possess adduct correlations

6 features were removed based on the screening *m/z* values in Column J from MS2 fragments in Column Q

Peak table processed by MetaboFR: Cluster-1569 contained 14 features, whereas 6 fragments were screened out and removed. Features flagged with adduct correlations were defined as top-rated features, while features with no adducts were defined as second-rated features.
