# Supplementary material for: Computational Workflow to Study the Diversity of Secondary Metabolites in Fourteen Different Isatis Species
Source: Cells. 2022 Mar 6;11(5):907. doi: 10.3390/cells11050907 (PMC8909408; doi:10.3390/cells11050907)
Supplement: Supplementary file 1 [file cells-11-00907-s001.zip › Supplementary File.pdf]

Supplementary File

Computational workflow to study the diversity of secondary metabolites in fourteen different *Isatis* species

Sample preparation and GC-MS analysis

Table S2. The information of the source of the seeds (14 species).

Figure S1. The phenotypes of 14 *Isatis* species.

Figure S2. Structural information of metabolites for li-1~66.

Figure S3. Structural information of metabolites for li-67~121.

Figure S4. Structural information of metabolites for li-122~170.

Figure S5. Structural information of metabolites for li-171~218.

Figure S6. Structural information of metabolites for li-219~269.

Table S3. The detailed information of 74 reference standards.

Table S4. An in-house library covers 269 metabolites from phytochemical research (up to 2020) in the whole *Isatis* L. genus.

Table S7. Validation results of detection of 73 reference standards by application of feature-rating rule.

Figure S7. Investigation of different chromatographic columns for achieving better separation and peak capacity (*Ind* as an example).

Figure S8. Investigation of different mobile phase additives for achieving better ionization (*Ind* as an example).

Figure S9. A standard mixture contains 12 purity compounds with different chemical classifications for the demonstration of mass features and adducts types before the large-scale acquisition.

Figure S10. The operation of detailed manual annotation of each feature in each cluster.

Figure S11. The relative content of indole compound by applying GC-MS among 12 species.

Sample preparation and GC-MS analysis

An amount of 10 mg of fresh samples was prepared using ultrasound extraction in 4 mL MeOH for 30 min. Then, the supernatant was dried under nitrogen-blow, which was redissolved in 1 mL cyclohexane, and kept in sealed vials at 4 °C until GC-MS analysis. GC–MS analysis was performed on Clarus 580 Gas Chromatograph (Perkin-Elmer, USA) equipped with an Elite-5 MS capillary column (30 m length, 0.25 mm internal diameter and 0.25 µm film thickness) and coupled with a SQ-8 MS detector. 0.1 µl volume of essential oil was injected. The oven temperature was programmed at 60 °C then increased to 220 °C at a rate of 3 °C per min and the final temperature was kept for 7 min at 220 °C. Helium at a flow rate of 1 ml/min was used as a carrier gas. The injector and transfer interface temperature was kept at 250 °C. Mass spectra were recorded at 70 electron volt. The source temperature was kept at 250 °C.

Table S2 The information of the source of the seeds (14 species).

Species Name	Collection location
Isatis indigotica Fort. (*ind*)	Shanghai, China
Isatis buschiana (*bus*)	North Korea
Isatis cappadocica (*cap*)	Turkey
Isatis cappadocica subsp. Steveniana (*cap*S)	Iran
Isatis costata C. A. Mey. (*cos*C)	Xinjiang, China
Isatis tinctoria L.(*tin*)	Germany
Isatis tinctoria L. var. *tinctoria* (*tin*V)	Xinjiang, China
Isatis japonica Miq. (*jap*)	Pyongyang, North Korea
*Isatis lusitanica*L. (*lus*)	Israel
Isatis glauca Aucherex Boiss. (*gla*A)	Israel
Tetraploid *Isatis indigotica* Fort. (4n=28), (4*ind*)	Shanghai, China
Isatis oblongata DC. (*obl*)	Xinjiang, China
Isatis violascens Bunge. (*vio*)	Xinjiang, China
Isatis minima Bunge. (*min*)	Xinjiang, China

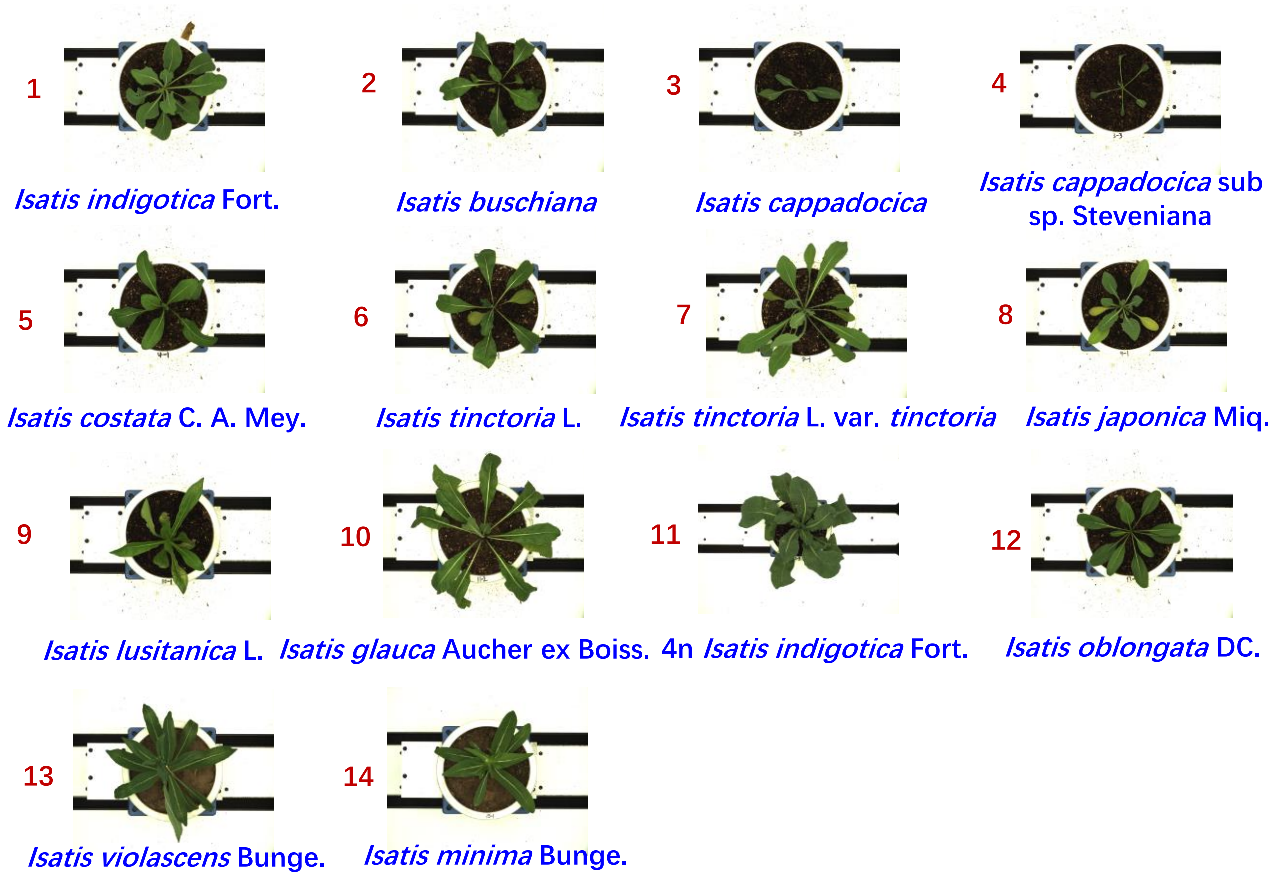

FigureS1. The phenotypes of 14 *Isatis* species.

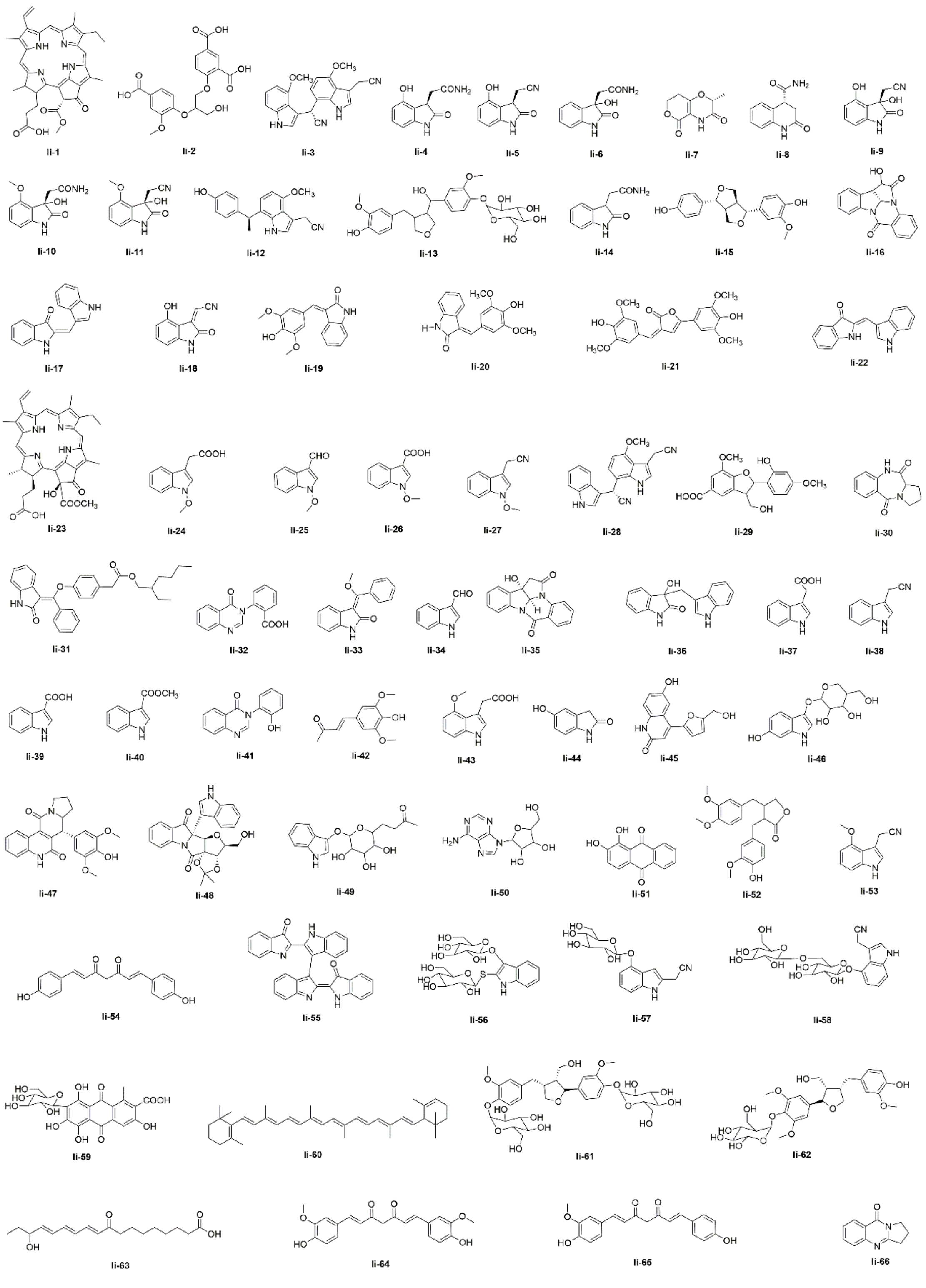

Figure S2. Structural information of metabolites for li-1~66.

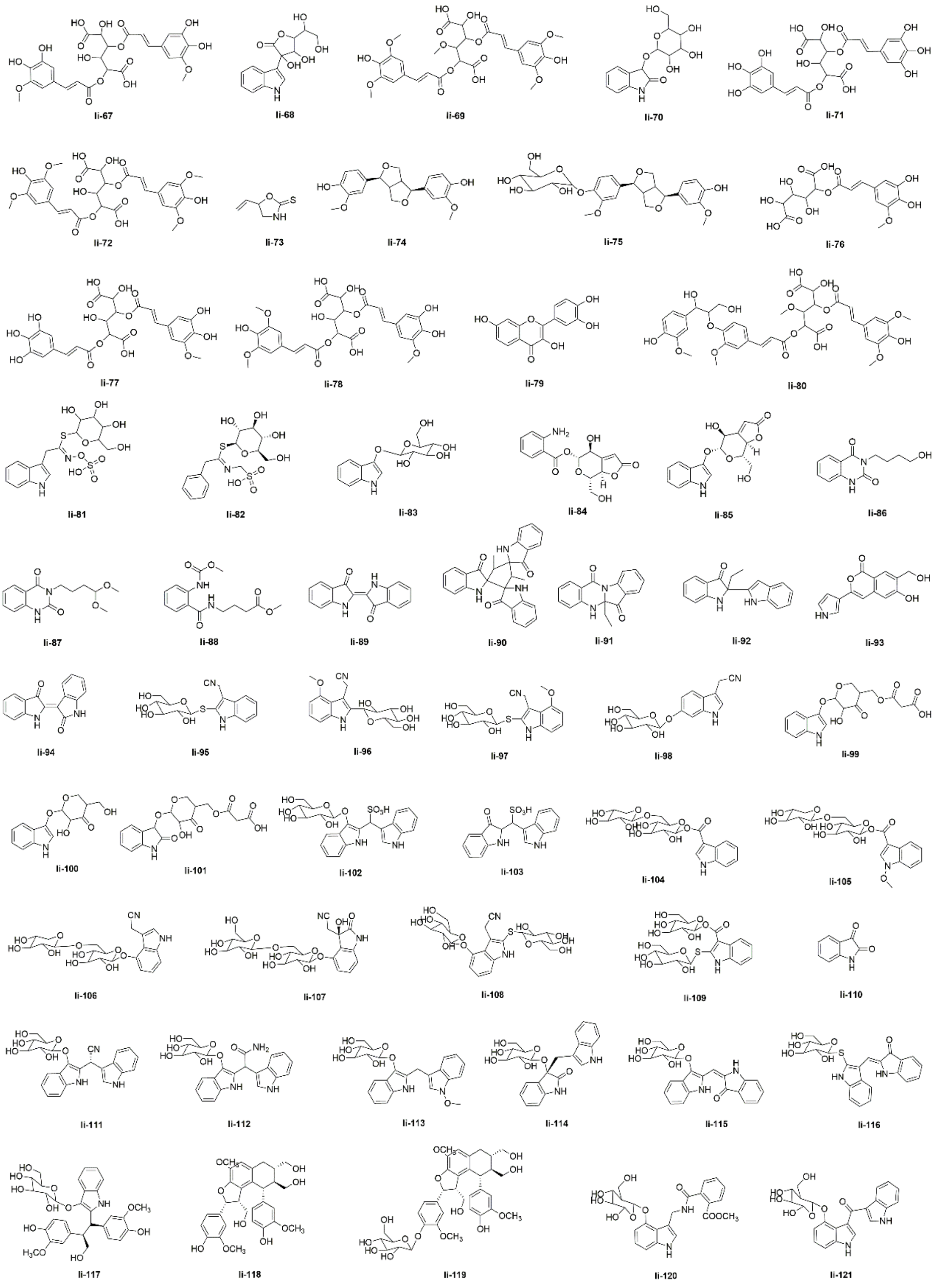

Figure S3. Structural information of metabolites for li-67~121.

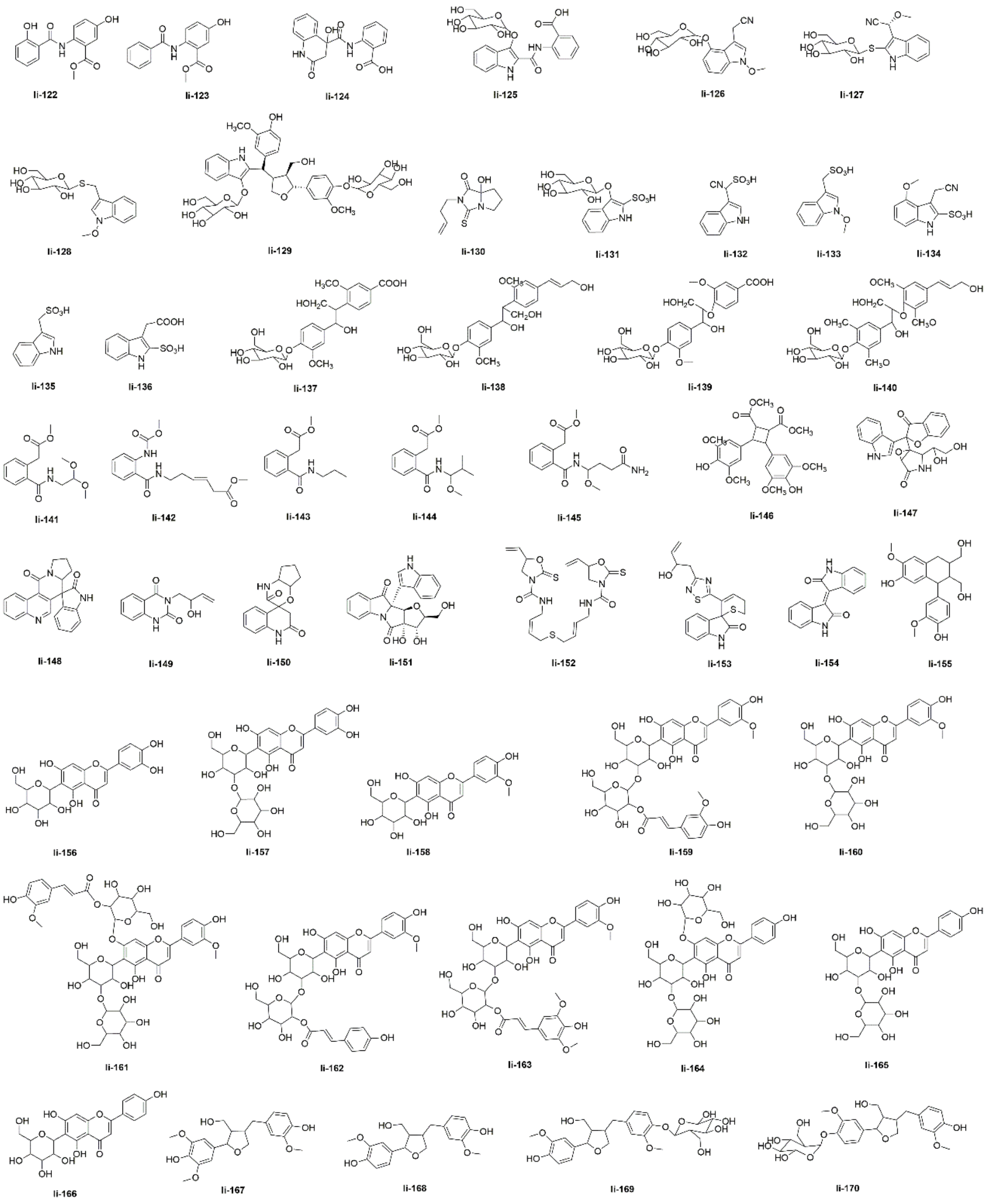

Figure S4. Structural information of metabolites for li-122~170.

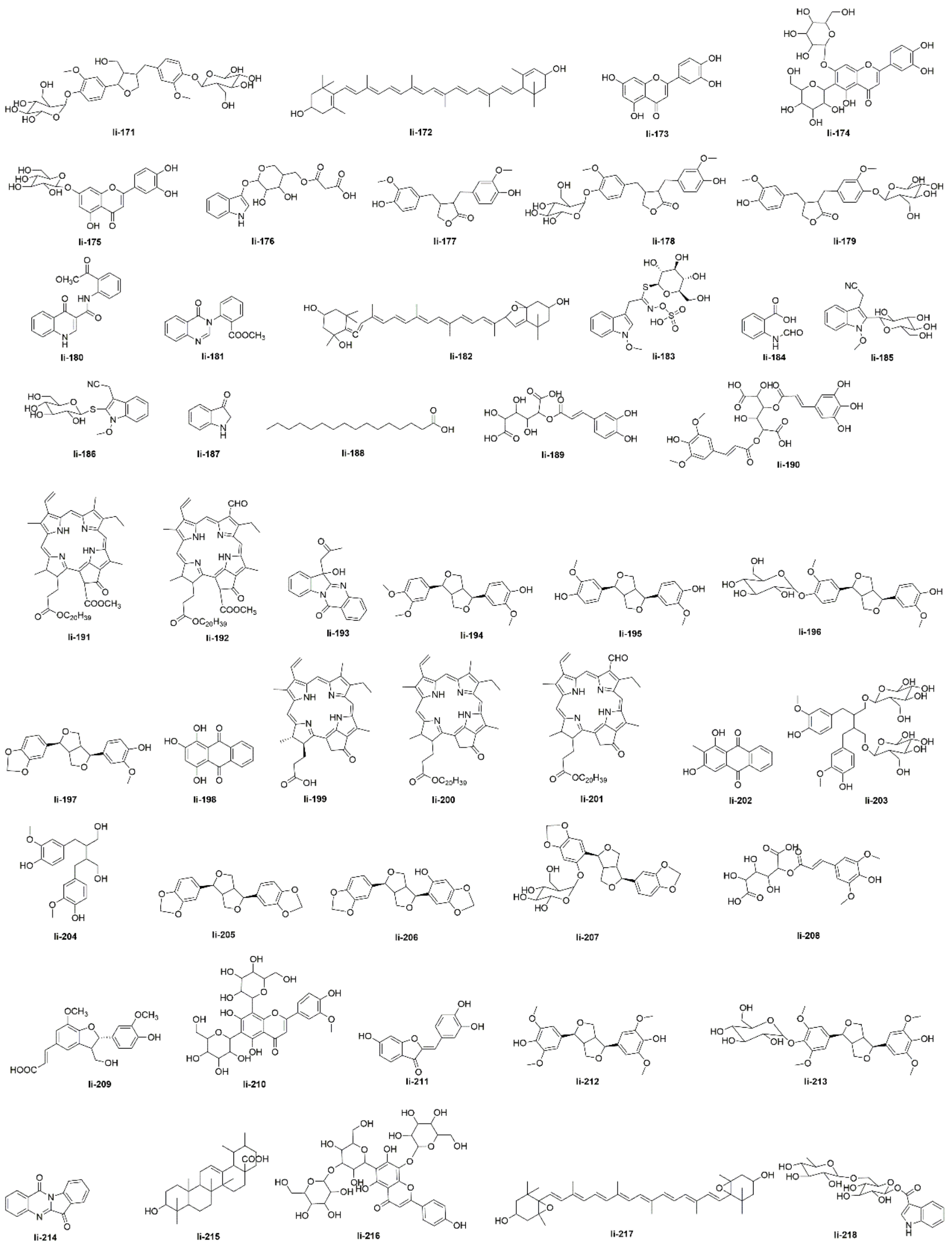

Figure S5. Structural information of metabolites for li-171~218.

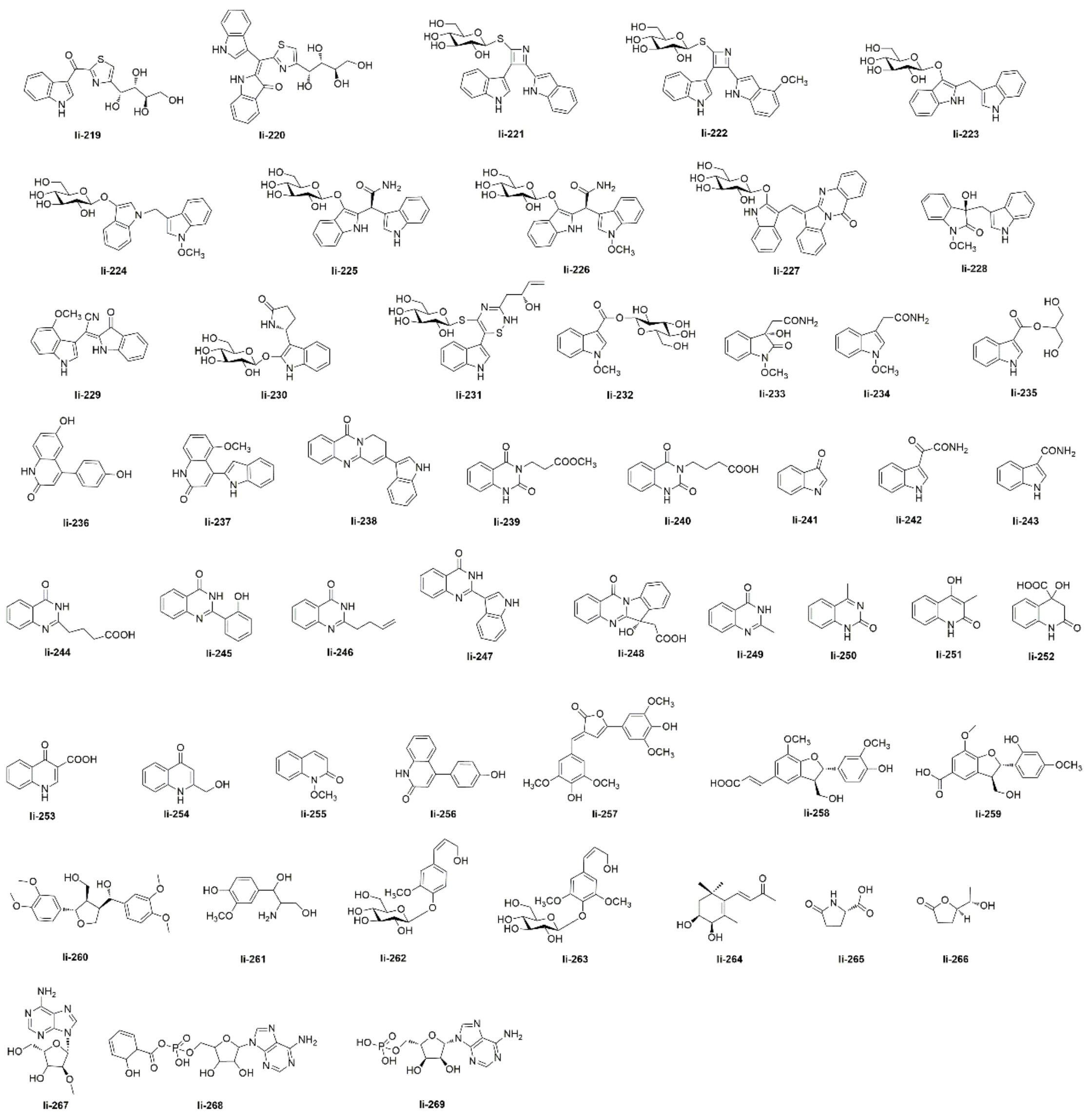

Figure S6. Structural information of metabolites for li-219~269.

Table S3 The detailed information of 74 reference standards.

No.	Name	Formula	CAS
1	Benzoic acid	$C_7H_6O_2$	65-85-0
2	Vanillin	$C_8H_8O_3$	121-33-5
3	3,4-Dihydroxybenzoic acid	$C_7H_6O_4$	99-50-3
4	Benzoyleneurea	$C_8H_6N_2O_2$	86-96-4
5	L-Phenylalanine	$C_9H_{11}NO_2$	63-91-2
6	Gallic acid	$C_7H_6O_5$	149-91-7
7	L(+)-Arginine	$C_6H_{14}N_4O_2$	74-79-3
8	Caffeic acid	$C_9H_8O_4$	331-39-5
9	D-Mannose	$C_6H_{12}O_6$	3458-28-4
10	Scopoletin	$C_{10}H_8O_4$	92-61-5
11	Ferulic Acid	$C_{10}H_{10}O_4$	1135-24-6
12	3-Hydroxy-4-methoxycinnamic acid	$C_{10}H_{10}O_4$	537-73-5
13	Wogonin	$C_{16}H_{12}O$	632-85-9
14	L-Tryptophan	$C_{11}H_{12}N_2O_2$	73-22-3
15	Isofraxidin	$C_{11}H_{10}O_5$	486-21-5
16	Sulbactam	$C_8H_{11}NO_5S$	68373-14-8
17	Gemcitabine	$C_9H_{11}F_2N_3O_4$	95058-81-4
18	Aloe emodin	$C_{15}H_{10}O_5$	481-72-1
19	Naringenin	$C_{15}H_{12}O_5$	480-41-1
20	Acacetin	$C_{16}H_{12}O_5$	480-44-4
21	Fludarabine	$C_{10}H_{12}FN_5O_4$	21679-14-1
22	3-Indoxyl-beta-D-glucopyranoside	$C_{14}H_{17}NO_6$	487-60-5
23	Hispidulin	$C_{16}H_{12}O_6$	1447-88-7
24	Salidroside	$C_{14}H_{20}O_7$	10338-51-9
25	Mycophenolic acid	$C_{17}H_{20}O_6$	24280-93-1
26	Gliclazide	$C_{15}H_{21}N_3O_3S$	21187-98-4
27	Tricin	$C_{17}H_{14}O_7$	520-32-1
28	Neochlorogenic acid	$C_{16}H_{18}O_9$	906-33-2
29	Cryptochlorogenic acid	$C_{16}H_{18}O_9$	905-99-7
30	Chlorogenic acid	$C_{16}H_{18}O_9$	327-97-9
31	Pinoresinol	$C_{20}H_{22}O_6$	487-36-5
32	Prednisolone	$C_{21}H_{28}O_5$	50-24-8
33	Loganin	$C_{17}H_{26}O_{10}$	18524-94-2
34	Icotinib	$C_{22}H_{21}N_3O_4$	610798-31-7
35	4,11-Diethyl-4,9-dihydroxy-1H-pyrano[3',4':6,7]indolizino[1,2-b]quinoline-3,14(4H,12H)-dione	$C_{22}H_{20}N_2O_5$	130144-34-2
36	Erlotinib	$C_{22}H_{23}N_3O_4$	183321-74-6
37	3β,5,14-Trihydroxy-5β-bufa-20,22-dienolide	$C_{24}H_{34}O_5$	472-26-4
38	2,3,5,4′-Tetrahydroxy stilbene-2-O-β-D-glucoside	$C_{20}H_{22}O_9$	82373-94-2
39	Liquiritin	$C_{21}H_{22}O_9$	551-15-5
40	Sec-O-glucosylhamaudol	$C_{21}H_{26}O_{10}$	80681-44-3
41	Cynaroside	$C_{21}H_{20}O_{11}$	5373-11-5

42	Astragalin	$C_{21}H_{20}O_{11}$	480-10-4
43	Oleanic acid	$C_{30}H_{48}O_3$	508-02-1
44	Oxytetracycline	$C_{22}H_{24}N_2O_9$	79-57-2
45	Hyperoside	$C_{21}H_{20}O_{12}$	482-36-0
46	Maslinic acid	$C_{30}H_{48}O_4$	4373-41-5
47	Corosolic acid	$C_{30}H_{48}O_4$	4547-24-4
48	Paeoniflorin	$C_{23}H_{28}O_{11}$	23180-57-6
49	Asiatic acid	$C_{30}H_{48}O_5$	464-92-6
50	Caudatin	$C_{28}H_{42}O_7$	38395-02-7
51	Baohuoside I	$C_{27}H_{30}O_{10}$	113558-15-9
52	Isochlorogenic acid C	$C_{25}H_{24}O_{12}$	57378-72-0
53	Ganoderic acid C2	$C_{30}H_{46}O_7$	98296-48-1
54	Forsythin	$C_{27}H_{34}O_{11}$	487-41-2
55	Arctiin	$C_{27}H_{34}O_{11}$	7770-78-7
56	Epirubicin	$C_{27}H_{29}NO_{11}$	56420-45-2
57	Ceritinib (LDK378)	$C_{28}H_{36}ClN_5O_3$ s	1032900-25-6
58	Polygalaxanthone III	$C_{25}H_{28}O_{15}$	162857-78-5
59	Procyandin B3	$C_{30}H_{26}O_{12}$	23567-23-9
60	Etoposide	$C_{29}H_{32}O_{13}$	33419-42-0
61	Vicenin	$C_{27}H_{30}O_{15}$	23666-13-9
62	Saponarin	$C_{27}H_{30}O_{15}$	20310-89-8
63	Rutin	$C_{27}H_{30}O_{16}$	153-18-4
64	Hesperidin	$C_{28}H_{34}O_{15}$	520-26-3
65	Ritonavir	$C_{37}H_{48}N_6O_5S_2$	155213-67-5
66	Timosaponin A-III	$C_{39}H_{64}O_{13}$	41059-79-4
67	Heterophyllin B	$C_{40}H_{58}N_8O_8$	145459-19-4
68	Saikosaponin A	$C_{42}H_{68}O_{13}$	20736-09-8
69	Epmedin C	$C_{39}H_{50}O_{19}$	110642-44-9
70	3-[(6-Deoxy-3-O-beta-D-glucopyranosyl-alpha-L-mannopyranosyl)oxy]-7-(beta-D-glucopyranosyloxy)-5-hydroxy-2-(4-methoxyphenyl)-8-(3-methyl-2-buten-1-yl)-4H-1-benzopyran-4-one	$C_{39}H_{50}O_{20}$	140147-77-9
71	Paclitaxel	$C_{47}H_{51}NO_{14}$	33069-62-4
72	Timosaponin BII	$C_{45}H_{76}O_{19}$	136656-07-0
73	Asiaticoside	$C_{48}H_{78}O_{19}$	16830-15-2
74	p-Hydroxybenzaldehyde	$C_7H_6O_2$	123-08-0

Table S4. An in-house library covers 269 metabolites from phytochemical research (up to 2020) in the whole *Isatis* L. genus.

**Code**	**Name**	**Formula**
Ii-1	pheophorbide a	$C_{35}H_{36}N_4O_5$
Ii-2	(-)-(R)- and(+)-(S)-isatindigotrioic acid	$C_{19}H_{18}O_{10}$
Ii-3	(-)-(R)-2-(3-cyanomethyl-4-methoxy-1H-indol-7-yl)-2-(4-methoxy-1H-indol-3-yl) acetonitrile	$C_{22}H_{18}N_4O_2$

Ii-4	(-)-(R)-2-(4-hydroxy-2-oxoindolin-3-yl) acetamide	$C_{10}H_{10}N_2O_3$
Ii-5	(-)-(R)-2-(4-hydroxy-2-oxoindolin-3-yl)-acetonitrile	$C_{10}H_{18}N_2O_2$
Ii-6	(-)-(S)-2-(3-hydroxy-2-oxoindolin-3-yl) acetamide	$C_{10}H_{10}N_2O_3$
Ii-7	(+)-(R)-2-methyl-7,8-dihydropyrano [4,3-b]-[1,4]oxazine-3,5(2H,4H)-dione	$C_8H_9NO_4$
Ii-8	(+)-(R)-2-oxo-1,2,3,4-tetrahydroquino-line-4-carboxamide	$C_{10}H_{10}N_2O_2$
Ii-9	(+)-(S)-2-(3,4-dihydroxy-2-oxoindolin-3-yl) acetonitrile	$C_{10}H_8N_2O_3$
Ii-10	(+)-(S)-2-(3-hydroxy-4-methoxy-2-oxoindolin-3-yl) acetamide	$C_{11}H_{12}N_2O_4$
Ii-11	(+)-(S)-2-(3-hydroxy-4-methoxy-2-oxoindolin-3-yl) acetonitrile	$C_{11}H_{10}N_2O_3$
Ii-12	(+)-(S)-2-{7-[1-(4-hydroxyphenyl)-ethyl]-4-methoxy-1H-indol-3-yl} acetonitrile	$C_{19}H_{18}N_2O_2$
Ii-3	(+)-larch rosin-4-O-B-D-glucoside	$C_{26}H_{34}O_{11}$
Ii-14	(±)-2-(2-oxoindolin-3-yl) acetamide	$C_{10}H_{10}N_2O_2$
Ii-15	(1S,2R,5S,6R)-2-(4-hydroxyphenyl)-6-(3-methoxy-4-hydroxyphenyl)-3,7-dioxab icyclo[3.3.0]octane	$C_{19}H_{20}O_5$
Ii-16	(2R,3R)-3-hydroxy-2Hpyrrolo[2,3-b]indolo[5,5a,6-b,a]quinazoline-9(8H),7'-dion e	$C_{17}H_{12}N_2O_3$
Ii-17	(2E)-2-(1H-indol-3ylmethylidene)-1,2-dihydro-3H-indol-3-one	$C_{17}H_{12}N_2O$
Ii-18	(E)-2-(4-hydroxy-2-oxoindolin-3-ylidene) acetonitrile	$C_{10}H_{16}N_2O_2$
Ii-19	(E)-3-(3',5'-dimethoxy-4'-hydroxybenzylidene)-2-indolinone.	$C_{17}H_{15}NO_4$
Ii-20	(E)-3-(30,50-dimethoxy-40-hydroxy-benzylidene)-2-indolinone	$C_{17}H_{15}NO_4$
Ii-21	(E)-5-(4-hydroxy-3,5-dimethoxyphenyl)-3-[(4-hydroxy-3,5-dimethoxyphenyl)-m ethylene]-2(3H)-furanone	$C_{21}H_{20}O_8$
Ii-22	(Z)-2-(1H-indol-3-ylmethyldene)-1,2-dihydro-3H-indol-3-one	$C_{17}H_{12}N_2O$
Ii-23	10-Hydroxy phaeophorbide	$C_{35}H_{36}N_4O_6$
Ii-24	1-methoxy-3-indoleacetic acid	$C_{11}H_{11}NO_3$
Ii-25	1-methoxy-3-indolecarbaldehyde	$C_{10}H_9NO_2$
Ii-26	1-methoxy-3-indoleformic acid	$C_{10}H_9NO_3$
Ii-27	1-methoxy-indole-3-acetonitrile	$C_{11}H_{10}N_2O$
Ii-28	2-(3-cyanomethyl-4-methoxy-1H-indol-7-yl)-2--(1H-indol-3-yl)acetonitrile	$C_{21}H_{16}N_4O$
Ii-29	2-(3-hydroxy-5-methoxyphenyl)-3-hydroxylmethyl-7-methoxy-2,3-dihydrobenz ofuran-5-carboxylic acid	$C_{18}H_{18}O_7$
Ii-30	2,3-dihydro-1 H-pyrrolo[2,1 -Cl[1 ,4]benzodiazepine-5,1 1(1 OH,1 I aH)-dione	$C_{12}H_{12}N_2O_2$
Ii-31	2-ethylhexyl 2-{4-[2-oxo-1, 2-dihydro-3H-indol-3-ylidene) (phenyl) methoxy] phenyl} acetate	$C_{31}H_{33}NO_4$
Ii-32	3-(2'-carboxyphenyl)-4(3H)-quinazolinone	$C_{15}H_{10}N_2O_3$
Ii-33	3-[(E)-methoxy (phenyl) methylidene]-1,3-dihydro-2H-indol-2-one	$C_{16}H_{13}NO_2$
Ii-34	3-aldehyde indole	C_9H_7NO
Ii-35	3-hydroxy-2H-pyrrolo[2,3-b]indolo[5,5a,6-b,a]quinazoline-9(8H),7′-dione	$C_{17}H_{12}N_2O_3$
Ii-36	3-hydroxy-2-ox-3,3’-diindolylmethane	$C_{17}H_{14}N_2O_2$
Ii-37	3-indoleacetic acid	$C_{10}H_9NO_2$
Ii-38	3-indoleacetonitrile	$C_{10}H_8N_2$
Ii-39	3-indoleformic acid	$C_9H_7NO_2$

Ii-40	3-indoleformic acid methyl ester	$C_{10}H_9NO_2$
Ii-41	4-(3H)-quinazolinone	$C_{14}H_{10}N_2O_2$
Ii-42	4-(4' -hydroxy-3' ,5' -dimethoxyphenyl)-3-buten-2-one	$C_{12}H_{14}O_4$
Ii-43	4-methoxy-3-indoleacetic acid	$C_{11}H_{11}NO_3$
Ii-44	5-Hydroxyoxindole	$C_8H_7NO_2$
Ii-45	6-hydroxy-4-(5-hydroxymethylfuran-2-yl)quinolin-2(1H)-one	$C_{14}H_{11}NO_4$
Ii-46	6-OH-ICOOGlc	$C_{14}H_{17}NO_6$
Ii-47	7-(3',5'-dimethoxy-4'-hydroxyphenyl)-7a,8,9,10-tetrahydroindolizino[7,6-c]quinoline-6,12(5H,7H)-dion	$C_{23}H_{22}N_2O_5$
Ii-48	9a,13a-Dihydroxylisopropylidenylisatisine A	$C_{25}H_{22}N_2O_6$
Ii-49	acetylindican	$C_{17}H_{21}NO_6$
Ii-50	adenosine	$C_{10}H_{13}N_5O_4$
Ii-51	alizarin	$C_{14}H_8O_4$
Ii-52	arctigentin	$C_{21}H_{24}O_6$
Ii-53	arvelexin	$C_{111}H_{10}N_2O$
Ii-54	bisdemethoxycurcumin	$C_{19}H_{16}O_4$
Ii-55	bisindigotin	$C_{32}H_{18}N_4O_2$
Ii-56	calanthoside	$C_{20}H_{27}NO_{11}S$
Ii-57	cappariloside A	$C_{16}H_{18}N_2O_6$
Ii-58	cappariloside B	$C_{22}H_{28}N_2O_{11}$
Ii-59	carminic acid	$C_{22}H_{20}O_{13}$
Ii-60	carotene	$C_{38}H_{54}$
Ii-61	clemastain B	$C_{32}H_{44}O_{16}$
Ii-62	conicaoside	$C_{27}H_{36}O_{12}$
Ii-63	corchorifatty acid B	$C_{18}H_{28}O_4$
Ii-64	curcumin	$C_{21}H_{20}O_6$
Ii-65	demethoxycurcumin	$C_{20}H_{18}O_5$
Ii-66	deoxyvasicinone	$C_{11}H_{10}N_2O$
Ii-67	diferuloylglucaric acid	$C_{26}H_{26}O_{16}$
Ii-68	dihydroascorbigen	$C_{14}H_{15}NO_6$
Ii-69	dinsinapoylmethoxyglucaric acid	$C_{29}H_{32}O_{16}$
Ii-70	dioxindoleglucoside	$C_{14}H_{17}NO_7$
Ii-71	di-p-coumaroylglucaric acid	$C_{24}H_{22}O_{16}$
Ii-72	disinapoylglucaric acid	$C_{28}H_{30}O_{16}$
Ii-73	epigoitrin	C_5H_7NOS
Ii-74	epipinoresinol	$C_{20}H_{22}O_6$
Ii-75	epipinoresinol-4-O-D-glucoside	$C_{26}H_{32}O_{11}$
Ii-76	feruloylglucaric acid	$C_{16}H_{18}O_{12}$
Ii-77	feruloyl p-coumaroylglucaric acid	$C_{25}H_{24}O_{16}$
Ii-78	feruloylsinapoylglucaric acid	$C_{27}H_{28}O_{16}$

Ii-79	fisetin	$C_{15}H_{10}O_6$
Ii-80	G(8-O-4)feruloylsinapolylgulcaric acid	$C_{38}H_{42}O_{19}$
Ii-81	glucobrassicin	$C_{16}H_{20}N_2O_9S_2$
Ii-82	glucotropaeolin	$C_{15}H_{21}NO_8S_2$
Ii-83	indican	$C_{14}H_{17}NO_6$
Ii-84	indiforine A	$C_{15}H_{15}NO_7$
Ii-85	indiforine B	$C_{16}H_{15}NO_6$
Ii-86	indiforine C	$C_{12}H_{14}N_2O_3$
Ii-87	indiforine D	$C_{14}H_{18}N_2O_4$
Ii-88	indiforine F	$C_{14}H_{18}N_2O_5$
Ii-89	indigo	$C_{16}H_{10}N_2O_2$
Ii-90	indigodole A	$C_{28}H_{23}N_3O_3$
Ii-91	indigodole B	$C_{17}H_{14}N_2O_2$
Ii-92	indigodole C	$C_{18}H_{16}N_2O$
Ii-93	indigotiisocoumarinA	$C_{14}H_{11}NO4$
Ii-94	indirubin	$C_{16}H_{10}N_2O_2$
Ii-95	indole-3-acetonitrile-2-S-β-d-glucopyranoside	$C_{16}H_{18}N_2O_5S$
Ii-96	indole-3-acetonitrile-4-methoxy-2-C-β-D-glucopyranoside	$C_{17}H_{20}N_2O_6$
Ii-97	indole-3-acetonitrile-4-methoxy-2-S-β-d-gluco- pyranoside	$C_{17}H_{20}N_2O_6S$
Ii-98	indole-3-acetonitrile-6-O-β-D-glucopyranoside	$C_{16}H_{18}N_2O_6$
Ii-99	isatan A	$C_{17}H_{17}NO_8$
Ii-100	isatan B	$C_{14}N_{15}NO_5$
Ii-101	isatan C	$C_{17}H_{17}NO_9$
Ii-102	isatibisindosulfonic acid A 3-O-β-D-glucopyranoside	$C_{23}H_{24}N_3O_9S$
Ii-103	isatibisindosulfonic acid B	$C_{17}H_{14}N_2O_4S$
Ii-104	isatigotindoledioside A	$C_{20}H_{25}NO_{11}$
Ii-105	isatigotindoledioside B	$C_{21}H_{27}NO_{12}$
Ii-106	isatigotindoledioside C	$C_{21}H_{26}N_2O_{10}$
Ii-107	isatigotindoledioside D	$C_{22}H_{28}N_2O_{13}$
Ii-108	isatigotindoledioside E	$C_{22}H_{28}N_2O_{11}S$
Ii-109	isatigotindoledioside F	$C_{21}H_{27}NO_{12}S$
Ii-110	isatin	$C_8H_5NO_2$
Ii-111	isatindigobisindoloside A	$C_{24}H_{23}N_3O_6$
Ii-112	isatindigobisindoloside C	$C_{24}H_{25}N_3O_7$
Ii-113	isatindigobisindoloside D	$C_{24}H_{26}N_2O_7$
Ii-114	isatindigobisindoloside E	$C_{23}H_{24}N_2O_7$
Ii-115	isatindigobisindoloside F	$C_{23}H_{22}N_2O_7$
Ii-116	isatindigobisindoloside G	$C_{23}H_{22}N_2O_6S$
Ii-117	isatindigodiphindoside	$C_{31}H_{35}NO_{11}$
Ii-118	isatindigosesquilignan A	$C_{30}H_{34}O_9$

Ii-119	isatindigosesquilignan B	$C_{36}H_{44}O_{14}$
Ii-120	isatindigoside A	$C_{24}H_{26}N_2O_9$
Ii-121	isatindigoside B	$C_{23}H_{22}N_2O_7$
Ii-122	isatindigoticamide A	$C_{15}H_{13}NO_5$
Ii-123	isatindigoticamide B	$C_{15}H_{13}NO_4$
Ii-124	isatindigoticoic acid A and epiisatindigoticoic acid A	$C_{17}H_{14}N_2O_5$
Ii-125	isatindigotindoloside A	$C_{22}H_{22}N_2O_9$
Ii-126	isatindigotindoloside B	$C_{17}H_{20}N_2O_7$
Ii-127	isatindigotindoloside C&D	$C_{17}H_{20}N_2O_6S$
Ii-128	isatindigotindoloside E	$C_{16}H_{21}NO_6S$
Ii-129	isatindolignanoside A	$C_{40}H_{49}NO_{17}$
Ii-130	isatindopyrromizol A and epiisatindopyrromizol A	$C_{10}H_{14}N_2O_2S$
Ii-131	isatindosulfonic acid A 3-O-β-D-glucopyranoside	$C_{14}H_{17}NO_9S$
Ii-132	isatindosulfonic acid B	$C_{10}H_8N_2O_3S$
Ii-133	isatindosulfonic acid C	$C_{10}H_{11}NO_4S$
Ii-134	isatindosulfonic acid D	$C_{11}H_{10}N_2O_4S$
Ii-135	isatindosulfonic acid E	$C_9H_9NO_3S$
Ii-136	isatindosulfonic acid F	$C_{19}H_9NO_5S$
Ii-137	isatioxyneolignoside A	$C_{24}H_{30}O_{12}$
Ii-138	isatioxyneolignosideE	$C_{26}H_{34}O_{11}$
Ii-139	isatioxyneolignosideB	$C_{24}H_{30}O_{13}$
Ii-140	isatioxyneolignoside X	$C_{28}H_{34}O_{14}$
Ii-141	isatiscaloids A	$C_{14}H_{19}NO_5$
Ii-142	isatiscaloids B	$C_{16}H_{20}N_2O_5$
Ii-143	isatiscaloids C	$C_{13}H_{17}NO_3$
Ii-144	isatiscaloids D	$C_{15}H_{21}NO_4$
Ii-145	isatiscaloids E	$C_{15}H_{20}N_2O_5$
Ii-146	isatiscycloneolignan A	$C_{24}H_{28}O_{10}$
Ii-147	isatisindigoticanine A	$C_{22}H_{18}N_2O_6$
Ii-148	isatisindigoticanine B	$C_{22}H_{17}N_3O_2$
Ii-149	isatisindigoticanine C	$C_{12}H_{12}N_2O_3$
Ii-150	isatisindigoticanine D	$C_{15}H_{16}N_2O_3$
Ii-151	isatisine A	$C_{22}H_{18}N_2O_6$
Ii-152	isatithioetherin A	$C_{20}H_{26}N_4O_4S_3$
Ii-153	isatithiopyrin B	$C_{18}H_{17}N_3O_2S_2$
Ii-154	isoindigo	$C_{16}H_{10}N_2O_2$
Ii-155	Isolariciresinol	$C_{20}H_{24}O_6$
Ii-156	isoorientin	$C_{21}H_{20}O_{11}$
Ii-157	isoorientin-3''-O-glucoside	$C_{27}H_{30}O_{16}$
Ii-158	isoscoparin	$C_{22}H_{22}O_{11}$

Ii-159	isoscoparin-3"-O-feruloylglucoside	$C_{38}H_{40}O_{19}$
Ii-160	isoscoparin-3"-O-glucoside	$C_{28}H_{32}O_{16}$
Ii-161	isoscoparin-3"-O-glucoside-7-O-glucoside	$C_{44}H_{50}O_{21}$
Ii-162	isoscoparin-3"-O-p-coumaroylglucoside	$C_{37}H_{38}O_{18}$
Ii-163	isoscoparin-3"-O-sinapoylglucoside	$C_{39}H_{42}O_{20}$
Ii-164	isovitexin-3"-O-glucoside-7-O-glucoside	$C_{33}H_{40}O_{20}$
Ii-165	isovitexin-3"-O-glucoside	$C_{27}H_{30}O_{15}$
Ii-166	isovitexin	$C_{21}H_{20}O_{10}$
Ii-167	justiciresinol	$C_{21}H_{26}O_{7}$
Ii-168	lariciresinol	$C_{20}H_{24}O_{6}$
Ii-169	lariciresinol-4'-O-D-glucopyranoside	$C_{26}H_{34}O_{11}$
Ii-170	lariciresinol-4-O-D-glucopyranoside	$C_{26}H_{34}O_{11}$
Ii-171	lariciresinol-diglucoside	$C_{32}H_{44}O_{16}$
Ii-172	lutein	$C_{38}H_{54}O_{2}$
Ii-173	luteolin	$C_{15}H_{10}O_{6}$
Ii-174	luteolin-6-C-glicoside-7-O-giucoside	$C_{27}H_{30}O_{16}$
Ii-175	luteolin-7-O-glucoside	$C_{21}H_{20}O_{11}$
Ii-176	malonylindican	$C_{17}H_{19}NO_{8}$
Ii-177	matairesinol	$C_{20}H_{22}O_{6}$
Ii-178	matairesinol-4'-O-D-glucopyranoside	$C_{26}H_{32}O_{11}$
Ii-179	matairesinol-4-O-D-glucopyranoside	$C_{26}H_{32}O_{11}$
Ii-180	methyl 2-(4-oxo-1,4-dihydroquinoline-3-carboxamido)-benzoate	$C_{18}H_{14}N_{2}O_{4}$
Ii-181	methyl 2-(4-oxoquinazolin-3(4H)-yl)benzoate	$C_{16}H_{12}N_{2}O_{3}$
Ii-182	neochrome	$C_{38}H_{54}O_{4}$
Ii-183	neoglucobrassicin	$C_{17}H_{22}N_{2}O_{10}S_{2}$
Ii-184	N-formylanthranilic acid	$C_{8}H_{7}NO_{3}$
Ii-185	N-methoxy-indole-3-acetonitrile-2-C-β-D-glucopyranoside	$C_{17}H_{20}N_{2}O_{6}$
Ii-186	N-methoxy-indole-3-acetonitrile-2-S-β-D-glucopyranoside	$C_{17}H_{20}N_{2}O_{6}S$
Ii-187	indoxyl	$C_{8}H_{7}NO$
Ii-188	palmitic acid	$C_{16}H_{32}O_{2}$
Ii-189	p-coumaroylglucaric acid	$C_{15}H_{16}O_{11}$
Ii-190	p-coumaroylsinapoylglucaric acid	$C_{26}H_{26}O_{16}$
Ii-191	phaeophytin a	$C_{55}H_{74}N_{4}O_{5}$
Ii-192	phaeophytin b	$C_{55}H_{72}N_{4}O_{6}$
Ii-193	phaitanthrin A and epiphaitanthrin A	$C_{18}H_{14}N_{2}O_{3}$
Ii-194	phillygenin	$C_{21}H_{24}O_{6}$
Ii-195	pinoresinol	$C_{20}H_{22}O_{6}$
Ii-196	pinoresinol 4-O-D-glucopyranoside	$C_{26}H_{32}O_{11}$
Ii-197	piperitol	$C_{20}H_{20}O_{6}$
Ii-198	purpurin	$C_{14}H_{8}O_{5}$

Ii-199	pyrophaeophorbide a	$C_{33}H_{34}N_4O_3$
Ii-200	pyrophaeophytin a	$C_{53}H_{72}N_4O_3$
Ii-201	pyrophaeophytin b	$C_{53}H_{70}N_4O_4$
Ii-202	rubiadin	$C_{15}H_{10}O_4$
Ii-203	secoisolariciresinol	$C_{20}H_{26}O_6$
Ii-204	secoisolariciresinoldiglucoside	$C_{32}H_{46}O_{16}$
Ii-205	sesamin	$C_{20}H_{18}O_6$
Ii-206	sesaminol	$C_{20}H_{18}O_7$
Ii-207	sesaminol-2’-O-D-glucose	$C_{26}H_{28}O_{12}$
Ii-208	sinapoylglucaric acid	$C_{17}H_{20}O_{12}$
Ii-209	spicatolignan B	$C_{20}H_{20}O_7$
Ii-210	stellarin-2	$C_{28}H_{32}O_{16}$
Ii-211	sulfuretin	$C_{15}H_{10}O_5$
Ii-212	syringaresinol	$C_{22}H_{26}O_8$
Ii-213	syringaresinol-4-O-D-glucoside	$C_{28}H_{36}O_{13}$
Ii-214	tryptanthrin	$C_{15}H_8N_2O_2$
Ii-215	ursolic acid	$C_{30}H_{48}O_3$
Ii-216	vicenin-2	$C_{27}H_{30}O_{15}$
Ii-217	violaxanthin	$C_{38}H_{54}O_4$
Ii-218	α-L-rhamnopyranosyl-(1→6)-β-D-glucopyranosyl 3-indolecarbonate	$C_{21}H_{27}NO_{11}$
Ii-219	isatisindigoticanine H	$C_{16}H_{16}N_2O_5S$
Ii-220	isatisindigoticanine I	$C_{24}H_{21}N_3O_5S$
Ii-221	isatindigosideF	$C_{25}H_{23}N_3O_5S$
Ii-222	isatindigoside G	$C_{26}H_{25}N_3O_6S$
Ii-223	isatindigoside H	$C_{23}H_{24}N_2O_6$
Ii-224	isatindigoside I	$C_{24}H_{26}N_2O_7$
Ii-225	isatindigoside D	$C_{24}H_{25}N_3O_7$
Ii-226	isatindigoside J	$C_{25}H_{27}N_3O_8$
Ii-227	isatindigoside E	$C_{30}H_{25}N_3O_7$
Ii-228	isatisindigoticanine J	$C_{18}H_{16}N_2O_3$
Ii-229	isatisindigoticanine K	$C_{19}H_{13}N_3O_2$
Ii-230	isatindigoside K	$C_{18}H_{22}N_2O_7$
Ii-231	isatindigoside L	$C_{21}H_{25}N_3O_6S_2$
Ii-232	isatindigoside M	$C_{16}H_{19}NO_8$
Ii-233	isatisindigoticanine L	$C_{11}H_{12}N_4O_4$
Ii-234	isatisindigoticanine M	$C_{11}H_{12}N_2O_2$
Ii-235	isatisindigoticanine N	$C_{12}H_{13}NO_4$
Ii-236	isatisindigoticanine E	$C_{15}H_{11}NO_3$
Ii-237	isatisindigoticanine F	$C_{18}H_{14}N_2O_2$
Ii-238	isatisindigoticanine G	$C_{20}H_{15}N_3O$

Ii-239	methyl 3-(2,4-dioxo-1,2-dihydroquinazolin-3(4H)-yl)propanoate	$C_{12}H_{12}N_2O_4$
Ii-240	4-(2,4-dioxo-1,2-dihydroquinazolin-3(4H)-yl)butanoic acid	$C_{12}H_{12}N_2O_4$
Ii-241	3H-indol-3-one	C_8H_5NO
Ii-242	(1H-Indol-3-yl)oxoacetamide	$C_{10}H_8N_2O_2$
Ii-243	1H-indole-3-carboxamide	$C_9H_8N_2O$
Ii-244	4-(4-oxo-3,4-dihydroquinazolin-2-yl)butanoic acid	$C_{12}H_{12}N_2O_3$
Ii-245	2-(2-hydroxyphenyl)-4(3H)-quinazolinone	$C_{14}H_{10}N_2O_2$
Ii-246	2-(but-3-en-1-yl)-4(3H)-quinazolinone	$C_{12}H_{12}N_2O$
Ii-247	2-(1H-indol-2-yl)-4(3H)-quinolinone	$C_{16}H_{11}N_3O$
Ii-248	cephalanthrin A	$C_{17}H_{12}N_2O_4$
Ii-249	2-methyl-4(3H)-quinazolinone	$C_9H_8N_2O$
Ii-250	4-methyl-1,2-dihydro-2-oxoquinazoline	$C_9H_8N_2O$
Ii-251	4-hydroxy-3-methyl-2(1H)-quinolone	$C_{10}H_9NO_2$
Ii-252	1,2,3,4-tetrahydro-4-hydroxy-quinolinecarboxylic acid	$C_{10}H_9NO_4$
Ii-253	4(1H)-quinolone-3-carboxylic acid	$C_{10}H_7NO_3$
Ii-254	2-(Hydroxymethyl)-4(1H)-quinolinone	$C_{10}H_9NO_2$
Ii-255	1-methoxy-2(1H)-quinolinone	$C_{10}H_9NO_2$
Ii-256	4-p-hydroxyphenyl-2(1H)-quinolinone	$C_{15}H_{11}NO_2$
Ii-257	(E)-5-(4-hydroxy-3,5-dimethoxyphenyl)-3-[(4-hydroxy-3,5-dimethoxyphenyl)-methylene]-2(3H)-furan one	$C_{21}H_{20}O_8$
Ii-258	spicatolignan B	$C_{20}H_{20}O_7$
Ii-259	2-(3'-hydroxy-5'-methoxyphenyl)-3-hydroxylmethyl-7-methoxy-2,3-dihydrobenzofuran-5-carboxylic acid	$C_{18}H_{18}O_7$
Ii-260	(7S,8R,7'S,8'R)-3,4,3',4'-tetramethoxy-9,7-dihydroxy-8.8',7.O.9'-lignan	$C_{22}H_{28}O_7$
Ii-261	2-amino-1-(4-hydroxy-3-methoxyphenyl)propane-1,3-diol	$C_{10}H_{15}NO_4$
Ii-262	cis-coniferin	$C_{16}H_{22}O_8$
Ii-263	cis-syringin	$C_{17}H_{22}O_{10}$
Ii-264	(E)-4-((3R,4S)-3,4-dihydroxy-2,6,6-trimethylcyclohex-1-en-1-yl)but-3-en-2-one	$C_{13}H_{20}O_3$
Ii-265	L-pyroglutamate	$C_5H_7NO_3$
Ii-266	(4R,5S)-5-hydroxyhexan-4-olide	$C_6H_{10}O_3$
Ii-267	2′-O-methyladenosine	$C_{11}H_{15}N_5O_4$
Ii-268	adenosine monophosphatederivatives	$C_{17}H_{20}N_5O_9P$
Ii-269	adenosine monophosphate	$C_{10}H_{14}N_5O_7P$

Table S7. Validation results of detection of 73 reference standards by application of feature-rating rule.

No.	RT (min)	*m/z*	Identification	Adduct types	Most intense
1	0.869	173.1046	L(+)-Arginine	$[M-H]^-$	$[M-H]^-$
2	0.921	215.0337	D-Mannose	$[M+Cl]^-$	$[M+Cl]^-$
3	0.95	298.0422	Gemcitabine	$[M+Cl]^-$, $[M+NO_3-H]^-$, $[M+COOH]^-$	$[M+Cl]^-$
4	1.55	169.0149	Gallic acid	$[M-H]^-$	$[M-H]^-$
5	2.104	164.0721	L-Phenylalanine	$[M-H]^-$	$[M-H]^-$
6	2.2	320.0567	Fludarabine	$[M+Cl]^-$	$[M+Cl]^-$
7	2.469	153.0202	3,4-Dihydroxybenzoic acid	$[M-H]^-$	$[M-H]^-$
8	2.59	353.0879	Neochlorogenic acid	$[M-H]^-$	$[M-H]^-$
9	2.838	345.1194	Salidroside	$[M+COOH]^-$	$[M+COOH]^-$
10	2.934	203.083	L-Tryptophan	$[M-H]^-$	$[M-H]^-$
11	3.031	577.135	Procyanidin B3	$[M-H]^-$	$[M-H]^-$
12	3.255	353.0883	Cryptochlorogenic acid	$[M-H]^-$	$[M-H]^-$
13	3.261	407.1123	Eleutheroside B	$[M+Cl]^-$, $[M+K-2H]^-$, $[M+COOH]^-$	$[M+Cl]^-$
14	3.408	353.0884	Chlorogenic acid	$[M-H]^-$	$[M-H]^-$
15	3.512	161.0371	Benzoyleneurea	$[M-H]^-$	$[M-H]^-$
16	3.555	330.0754	3-Indoxyl-beta-D-glucopyranoside	$[M+Cl]^-$, $[M+COOH]^-$	$[M+Cl]^-$
17	3.715	179.0352	Caffeic acid	$[M-H]^-$	$[M-H]^-$
18	3.825	593.1509	Vicenin	$[M-H]^-$	$[M-H]^-$
19	3.922	435.1514	Loganin	$[M+COOH]^-$	$[M+COOH]^-$
20	4.188	593.1506	Saponarin	$[M-H]^-$	$[M-H]^-$
21	4.255	459.1401	Oxytetracycline	$[M-H]^-$	$[M-H]^-$
22	4.409	441.0944	2,3,5,4′-Tetrahydroxy stilbene-2-O-β-D-glucoside	$[M+Cl]^-$, $[M-H]^-$	$[M+Cl]^-$
23	4.525	525.1608	Paeoniflorin	$[M+COOH]^-$	$[M+COOH]^-$
24	4.593	567.1343	Polygalaxanthone III	$[M-H]^-$	$[M-H]^-$
25	4.672	151.0403	Vanillin	$[M-H]^-$	$[M-H]^-$
26	4.798	609.1458	Rutin	$[M-H]^-$	$[M-H]^-$
27	4.942	463.0889	Hyperoside	$[M-H]^-$	$[M-H]^-$
28	5.021	191.0355	Scopoletin	$[M-H]^-$	$[M-H]^-$
29	5.045	441.0954	2,3,5,4′-Tetrahydroxy stilbene-2-O-β-D-glucoside	$[M+Cl]^-$	$[M+Cl]^-$
30	5.045	453.0964	Liquiritin	$[M+Cl]^-$, $[M+NO_3-H]^-$	$[M+Cl]^-$
31	5.088	193.051	Ferulic acid	$[M-H]^-$	$[M-H]^-$
32	5.106	483.0685	Cynaroside	$[M+Cl]^-$	$[M+Cl]^-$
33	5.231	221.046	Isofraxidin	$[M-H]^-$	$[M-H]^-$
34	5.335	193.051	3-Hydroxy-4-methoxycinnamic acid	$[M-H]^-$	$[M-H]^-$
35	5.536	447.0931	Astragalin	$[M-H]^-$	$[M-H]^-$
36	5.857	515.1192	Isochlorogenic acid C	$[M-H]^-$	$[M-H]^-$
37	5.891	645.158	Hesperidin	$[M+Cl]^-$	$[M+Cl]^-$

38	6.032	121.0296	Benzoic acid	$[M-H]^-$	$[M-H]^-$
39	6.644	919.4899	Timosaponin BII	$[M+Cl]^-$, $[M-H]^-$	$[M-H]^-$
40	6.644	426.1231	Icotinib	$[M+Cl]^-$, $[M+K-2H]^-$	$[M+Cl]^-$
41	6.884	329.0663	Tricin	$[M-H]^-$	$[M-H]^-$
42	6.982	579.2072	Forsythin	$[M+COOH]^-$	$[M+COOH]^-$
43	7.01	391.1303	4,11-Diethyl-4,9-dihydroxy-1H-pyrano[3',4':6,7]indolizino[1,2-b]quinoline-3,14(4H,12H)-dione	$[M-H]^-$	$[M-H]^-$
44	7.03	873.2579	3-[(6-Deoxy-3-O-beta-D-glucopyranosyl-alpha-L-mannopyranosyl)oxy]-7-(beta-D-glucopyranosyloxy)-5-hydroxy-2-(4-methoxyphenyl)-8-(3-methyl-2-buten-1-yl)-4H-1-benzopyran-4-one	$[M+Cl]^-$, $[M+NO_3-H]^-$	$[M+Cl]^-$
45	7.082	578.1428	Epirubicin	$[M-H]^-$	$[M-H]^-$
46	7.093	579.2067	Arctiin	$[M+COOH]^-$	$[M+COOH]^-$
47	7.272	993.4821	Asiaticoside	$[M+Cl]^-$	$[M+Cl]^-$
48	7.351	473.1222	Sec-O-glucosylhamaudol	$[M+Cl]^-$, $[M+COOH]^-$	$[M+Cl]^-$
49	7.408	395.1638	Prednisolone	$[M+Cl]^-$, $[M+K-2H]^-$	$[M+Cl]^-$
50	7.426	884.2804	Epmedin C	$[M+NO_3-H]^-$	$[M+NO_3-H]^-$
51	7.432	623.1536	Etoposide	$[M+Cl]^-$	$[M+Cl]^-$
52	7.66	428.1384	Erlotinib	$[M+Cl]^-$, $[M+K-2H]^-$	$[M+Cl]^-$
53	8.118	271.0619	Naringenin	$[M-H]^-$	$[M-H]^-$
54	8.241	553.2932	Ganoderic acid C2 (SH)	$[M+Cl]^-$, $[M+K-2H]^-$, $[M+NO_3-H]^-$	$[M+Cl]^-$
55	8.245	299.0564	Hispidulin	$[M-H]^-$	$[M-H]^-$
56	8.575	813.4068	Heterophyllin B	$[M+Cl]^-$, $[M+NO_3-H]^-$	$[M+Cl]^-$
57	8.652	447.2381	3β,5,14-Trihydroxy-5β-bufa-20,22-dienolide	$[M+COOH]^-$	$[M+COOH]^-$
58	9.828	341.1006	Mycophenolic acid	$[M-Na-2H]^-$	$[M-Na-2H]^-$
59	9.9	269.0483	Aloe emodin	$[M-H]^-$	$[M-H]^-$
60	10.409	283.061	Acacetin	$[M-H]^-$	$[M-H]^-$
61	10.529	815.4357	Saikosaponin A	$[M+Cl]^-$, $[M+COOH]^-$, $[M+NO_3-H]^-$	$[M+Cl]^-$
62	10.581	283.0608	Wogonin	$[M-H]^-$	$[M-H]^-$
63	10.893	322.1231	Gliclazide	$[M-H]^-$	$[M-H]^-$
64	10.971	513.1757	Baohuoside I	$[M-H]^-$	$[M-H]^-$
65	11.243	556.2142	Ceritinib	$[M-H]^-$	$[M-H]^-$
66	11.527	523.3182	Asiatic acid	$[M+Cl]^-$, $[M+K-2H]^-$	$[M+Cl]^-$
67	11.951	527.2603	Caudatin	$[M-H]^-$, $[M+Cl]^-$, $[M+K-2H]^-$	$[M+Cl]^-$
68	12.286	915.3181	Paclitaxel	$[M+NO_3-H]^-$	$[M+NO_3-H]^-$
69	12.796	765.3094	Ritonavir	$[M+COOH]^-$, $[M+NO_3-H]^-$	$[M+COOH]^-$
70	13.075	775.4041	Timosaponin A-III	$[M+Cl]^-$, $[M+NO_3-H]^-$	$[M+Cl]^-$
71	14.737	471.3481	Maslinic acid	$[M-H]^-$	$[M-H]^-$
72	14.917	471.3475	Corosolic acid	$[M-H]^-$	$[M-H]^-$

73	17.888	455.352	Oleanic acid	[M-H]-	[M-H]-

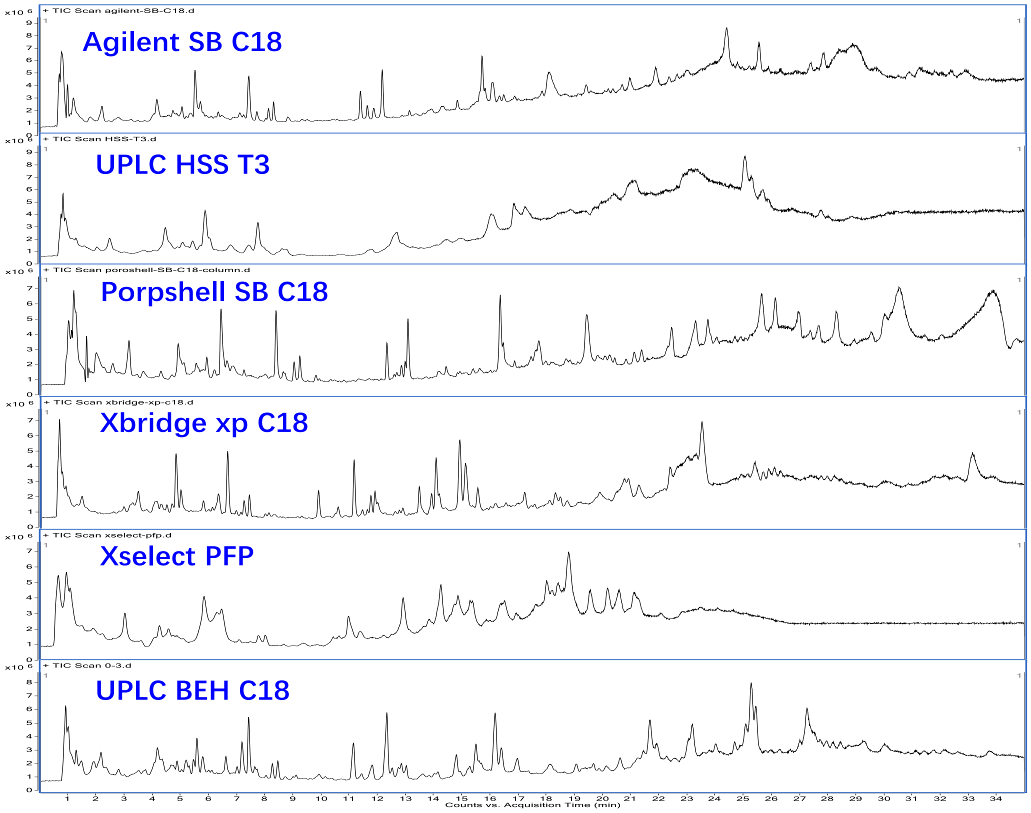

Figure S7. Investigation of different chromatographic columns for achieving better separation and peak capacity (*Ind* as an example).

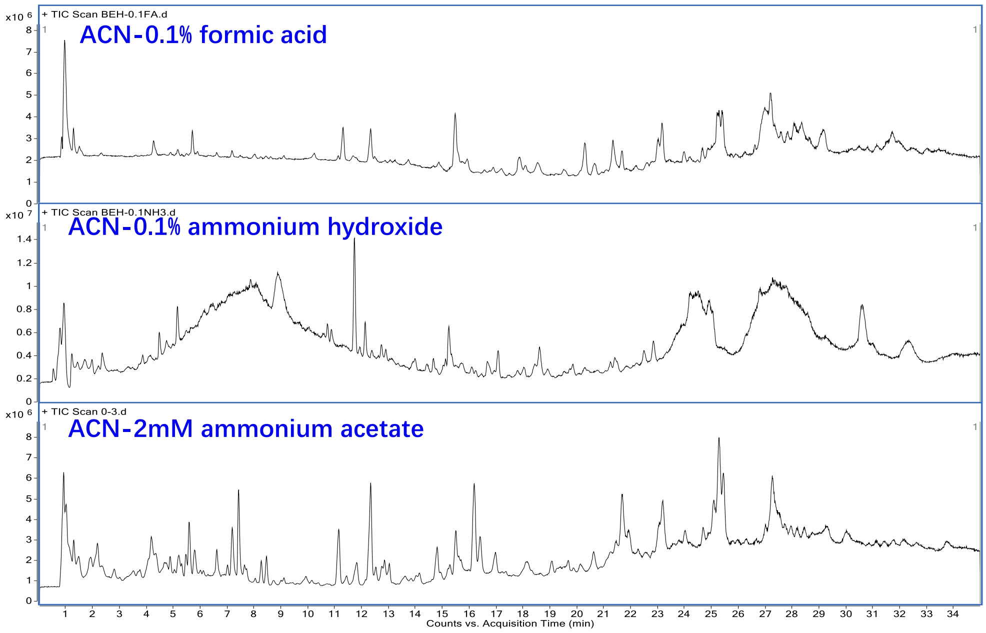

Figure S8. Investigation of different mobile phase additives for achieving better ionization (*Ind* as an example).

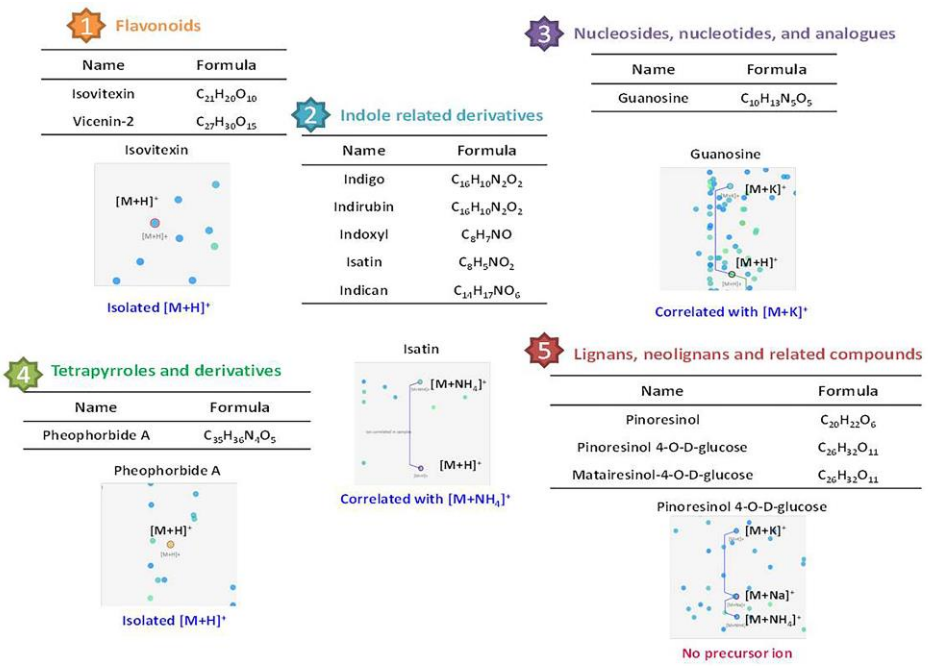

Figure S9. A standard mixture contains 12 purity compounds with different chemical classifications for the demonstration of *m/z* and adducts types before the large-scale acquisition.

Detailed manual annotation of each feature in feature table (4072)

Cluster-608

No.	RT (min)	*m/z*	Adduct type	Fragment	Correlation
1	4.00	359.1456	[M+H]+		6, 7
2	3.97	304.1651	[M+H]+		\
3	3.97	482.1256	[M+H]+		\
4	4.01	531.1681	[M+H]+		\
5	3.97	407.1307	[M+K]+		8
6	3.99	366.0599	[M+K]+		7
7	3.99	350.0847	[M+Na]+		6
8	3.98	386.2018	[M+NH4]+		5
9	3.97	344.0768	[M+H]+		\
10	3.97	554.2210	[M+H]+		\

→ INT (UnRECG); SRF (ID); SRF (ID); SRF (ID); TRF; TRF; TRF (most); TRF (most); SRF (UnID); SRF (UnID)

Correlated with 6 and 7 (Pearson correlation coefficient≥0.8).

INT (UnRECG) → Unknown adduct ion; dimers; trimers; etc.

SRF (ID) → Exhibit only quasi-molecular ion.

TRF / SRF (UnID) → Adduct correlations observed and ignore the MS^2 validation.

Cluster-1513

No.	RT (min)	*m/z*	Adduct type	Fragment	Correlation
1	7.12	531.1697	[M+H]+	Removed	4, 6, 8, 10
2	7.11	495.1366	[M+H]+		\
3	7.11	349.126	[M+Na]+		7
4	7.12	777.2216	[M+Na]+		1, 6, 8, 10
5	7.12	461.1986	[M+Na]+		9
6	7.12	793.1952	[M+K]+		1, 4, 8, 10
7	7.11	365.1007	[M+K]+		3
8	7.12	772.2654	[M+NH4]+		1, 4, 6, 10
9	7.15	456.24332	[M+NH4]+		5
10	7.11	786.27777	[M+H]+		1, 4, 6, 8

→ FRGM; SRF (ID); TRF; TRF; TRF; TRF; TRF (most); TRF (most); TRF (most); INT (UnRECG)

FRGM → Correlated with corresponding precursor ions. (Pearson correlation coefficient≥0.8).

TRF: top-rated feature ; TRF (most): most intense top-rated feature ; SRF (ID): identified second-rated feature;

SRF (UnID): Unidentified second-rated feature; FRGM: in-source fragment; INF (UnRECG): Unrecognized interference.

Figure S10. The operation of detailed manual annotation of each feature in each cluster.

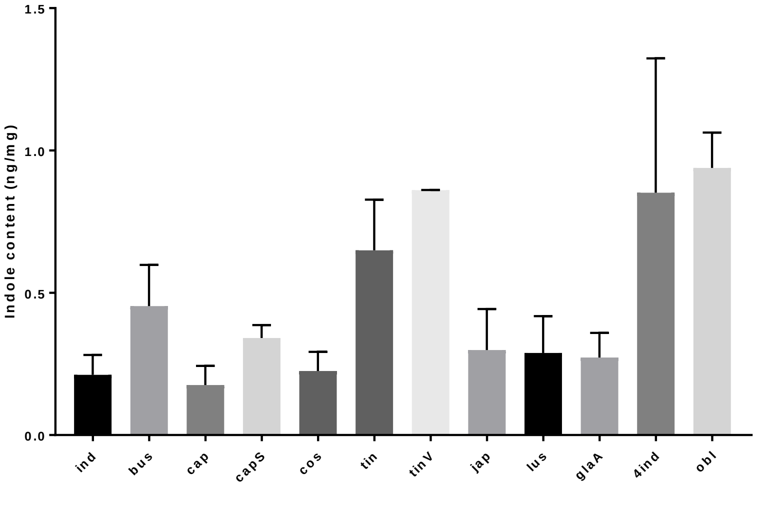

Figure S11. The relative content of indole compound by applying GC-MS among 12 species.
